# iSeq 100 for metagenomic pathogen screening in ticks

**DOI:** 10.1186/s13071-021-04852-w

**Published:** 2021-06-29

**Authors:** Ju Yeong Kim, Myung-hee Yi, Alghurabi Areej Sabri Mahdi, Tai-Soon Yong

**Affiliations:** 1 Department of Environmental Medical Biology, Institute of Tropical Medicine, Arthropods of Medical Importance Resource Bank, Yonsei University College of Medicine, Yonsei-ro 50-1, Seodaemun-gu, Seoul, 03722 South Korea; 2 Department of Global Health Security, Graduate School of Public Health, Yonsei University, Seoul, 03722 South Korea

**Keywords:** *Haemaphysalis longicornis*, iSeq 100, Next generation sequencing, *Rickettsia*, Vector-borne disease

## Abstract

**Background:**

Ticks are blood-sucking ectoparasites that play a pivotal role in the transmission of various pathogens to humans and animals. In Korea, *Haemaphysalis longicornis* is the predominant tick species and is recognized as the vector of pathogens causing various diseases such as babesiosis, borreliosis, rickettsiosis, and severe fever with thrombocytopenia syndrome.

**Methods:**

In this study, the targeted high-throughput sequencing of the 16S rRNA V4 region was performed using the state-of-the-art sequencing instrument, iSeq 100, to screen bacterial pathogens in *H. longicornis*, and the findings were compared with those using conventional PCR with specific primers. Microbiome analyses were performed with EzBioCloud, a commercially available ChunLab bioinformatics cloud platform. ANOVA-Like Differential Expression tool (ALDEx2) was used for differential abundance analysis.

**Results:**

*Rickettsia* spp. were detected in 16 out of 37 samples using iSeq 100, and this was confirmed using a PCR assay. In the phylogenetic analysis using *gltA* and *ompA* sequences of the detected *Rickettsia*, the highest sequence similarity was found with ‘*Candidatus* Rickettsia jingxinensis’ isolate Xian-Hl-79, ‘*Ca*. R. jingxinensis’ isolate F18, and ‘*Ca*. R. longicornii‘ isolate ROK-HL727. In the microbiome study, *Coxiella* AB001519, a known tick symbiont, was detected in all 37 tick samples. *Actinomycetospora chiangmaiensis* was more abundant in *Rickettsia*-positive samples than in *Rickettsia*-negative samples.

**Conclusions:**

In this study, iSeq 100 was used to investigate the microbiome of *H. longicornis*, and the potentially pathogenic *Rickettsia* strain was detected in 16 out of 37 ticks. We believe that this approach will aid in large-scale pathogen screening of arthropods to be used in vector-borne disease control programs.

**Graphical Abstract:**

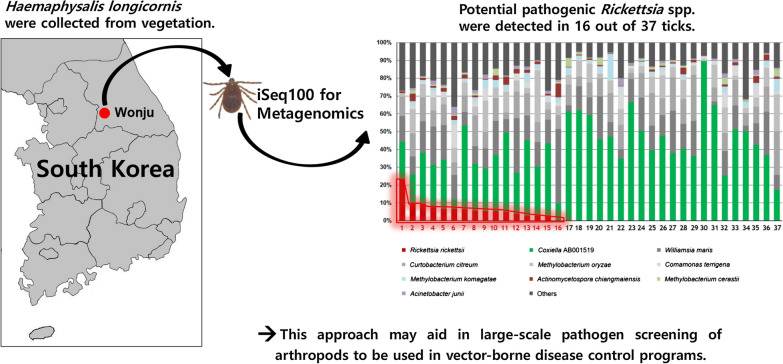

**Supplementary Information:**

The online version contains supplementary material available at 10.1186/s13071-021-04852-w.

## Background

Ticks are blood-sucking ectoparasites that play a pivotal role in the transmission of a variety of pathogens to humans and animals [1, 2]. Ticks harbor numerous bacterial, protozoal, and viral pathogens and transfer these into the host’s body when sucking blood, causing serious infectious diseases [3, 4]. Tick-borne diseases such as anaplasmosis, ehrlichiosis, borreliosis, babesiosis, and rickettsiosis represent emerging threats to public and animal health worldwide [5, 6]. Each tick species has preferred environmental conditions, which determine the geographic distribution of the tick and, consequently, the risk areas for tick-borne diseases [1].

Ixodid ticks (including *Haemaphysalis longicornis*) have been recognized as vectors of pathogens causing diseases such as babesiosis, borreliosis, rickettsiosis, and severe fever with thrombocytopenia syndrome (SFTS) in Korea [7–13]. *Rickettsia* spp. have been isolated from infected animals, ticks, and the blood of human patients in Korea [13–18].

The main advantage of next-generation sequencing (NGS) is that a wide array of known and unknown pathogens can be identified simultaneously, without the need for designing individual specific bacterial primers [19]. iSeq 100, a recently released instrument, is capable of processing a targeted NGS library with accuracy comparable to that of MiSeq, the most widely used instrument in microbiome studies [20]. iSeq 100 also features shortened sequencing workflow preparation time and a total sequencing run time of under 24 h. The reduced capital and maintenance costs are also advantages of iSeq 100 over MiSeq [20].

In this study, we used iSeq 100 to detect bacterial pathogens in *H. longicornis* and compared the pathogen detection rates to those of pathogen-specific PCR test. In addition, we identified the strain of the detected potential pathogens using PCR with specific primers.

## Methods

### Sample collection and processing

Ticks were collected from the vegetation by flagging in Wonju (Gangwon-do Province; 37.389545, 127.801770) in July 2015. Species identification of collected ticks was performed by examination under a dissecting microscope according to the method of Yamaguti et al. [21]. Only nymph stage ticks were used in the study. The surface of each tick was sterilized using alcohol before DNA extraction, and whole ticks were used for DNA extraction. DNA was extracted from each tick using a NucleoSpin DNA Insect kit (Macherey-Nagel, Düren, Germany) according to the manufacturer’s instructions, and stored at −20 °C until use.

### Illumina sequencing and bioinformatics

Total DNA was extracted using the NucleoSpin DNA Insect Kit (Macherey-Nagel, Düren, Germany) following the instructions of the manufacturer. The 16S rDNA V4 region was amplified by PCR using primers of 515F (5′-TCGTCGGCAGCGTCAGATG TGTATAAGAGACAGGTGCCAGCMGCCGCGGTAA-3′) and 806R (5′-GTCTCGTGGG CTCGGAGATGTGTATAAGAGACAGGGACTACHVGGGTWTCTAAT-3′) [22]. A limited-cycle (eight cycles) amplification step was performed to add multiplexing indices and Illumina sequencing adapters. Mixed amplicons were pooled, and the sequencing was performed with the Illumina iSeq 100 sequencing system according to the manufacturer’s instructions, utilizing an Illumina iSeq™ 100 i1 Reagent v2 kit (San Diego, CA, USA).

Processing raw reads started with a quality check and the filtering of low-quality (< Q25) reads by Trimmomatic ver. 0.321 [23]. After a quality control pass, sequence data were merged using the fastq_mergepairs command of VSEARCH version 2.13.42 with default parameters [24]. Primers were then trimmed with the alignment algorithm of Myers and Miller [25] at a similarity cut-off of 0.8. Non-specific amplicons that do not encode 16S rRNA were detected by nhmmer [26] in the HMMER software package ver. 3.2.1 with hmm profiles. Unique reads were extracted, and redundant reads were clustered with the unique reads by derep_fulllength command of VSEARCH2. The EzBioCloud 16S rRNA database [27] was used for taxonomic assignment using the usearch_global command of VSEARCH2, followed by more precise pairwise alignment [25]. Chimeric reads were filtered on reads with < 97% similarity by reference-based chimeric detection using the UCHIME algorithm [28] and the non-chimeric 16S rRNA database from EzBioCloud. After chimeric filtering, reads that were not identified to the species level (with < 97% similarity) in the EzBioCloud database were compiled, and the cluster_fast command was used to perform de novo clustering to generate additional operational taxonomic units (OTUs). Finally, OTUs with single reads (singletons) were omitted from further analysis. All subsequent analyses were performed with EzBioCloud, a commercially available ChunLab bioinformatics cloud platform for microbiome research (https://www.ezbiocloud.net/). The reads were normalized to 2500 to perform the analyses. We computed the Shannon index [29] and performed principal coordinate analysis (PCoA) [30]. The Wilcoxon rank-sum test was used to test the difference in the number of OTUs and the Shannon index between two groups. Significant differences in the relative abundances at the phylogenetic level between the two groups were assessed using the ANOVA-Like Differential Expression version 2 (ALDEx2) package and were visualized with the beeswarm package in R software (version 4.0.5) [31].

### PCR, sequencing, and phylogenetic analysis

To detect the partial sequences of 17 kDa antigen-encoding gene of *Rickettsia* spp. in ticks, nested PCR was performed with two primer pairs: Rr17k.Ip (5′-TTTACAAAATTCTAAAAACCAT-3′) and Rr17k.539n (5′-TCAATTCACAACTTGCCATT-3′), which amplify a 539 bp fragment, and Rr17k.90p (5′-GCTCTTGCAACTTCTATGTT-3′) and Rr17k.539n (5′-TCAATTCACAACTTGCCATT-3′), which amplify a 450 bp fragment [32]. Sequencing of *Rickettsia*-positive nested PCR amplicons was conducted by Bionics Co. (Seoul, Korea). To identify the species using *Rickettsia*-positive nested PCR amplicons, the *gltA* gene and *ompA* gene were PCR-amplified. The sequences of the primers were as follows: *gltA*, 5′-GGCTAATGAAGCGGTAATAAATATGCTT-3′ (forward) and 5′-TTTGCGACGGTATAC C CATAGC-3′ (reverse); *ompA*, 5′-CACYACCTCAACCGCAGC-3′ (forward), and 5′-AAAGT TATATTTCCTAAACCYGTATAAKTATCRGC-3′ (reverse) [33].

A BLAST search was used to compare the obtained sequence of the *gltA* gene and *ompA* gene to those available in GenBank (USA). The obtained sequences were compared for similarity to sequences deposited in GenBank using BLAST. Gene sequences, excluding the primer regions, were aligned using the multisequence alignment program in Clustal Omega (Cambridgeshire, UK) [34]. The phylogenetic trees were constructed by Molecular Evolution Genetics Analysis (MEGA X) software using the maximum-likelihood method and employing the Tamura-nei model of nucleotide substitution with 1000 bootstrap replications [35].

## Results

### The microbiome composition of ticks analyzed with iSeq 100

**Fig. 1:**
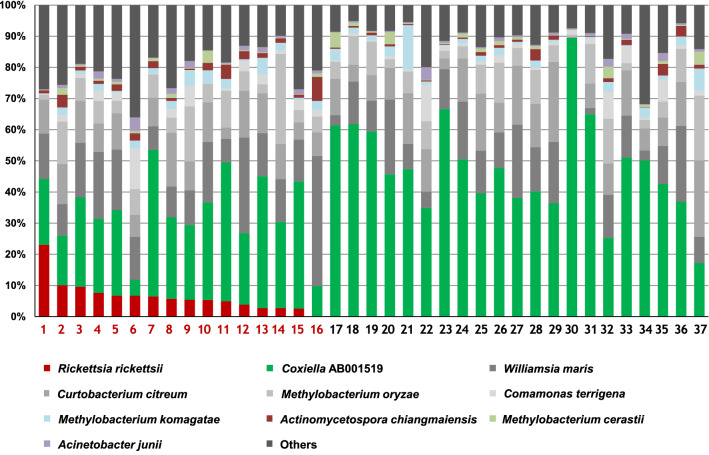
The microbiome composition of each tick at the species level (*n* = 37). Species accounting for more than 1% of total reads are shown. Tick samples 1–16 (red) were found to harbor *Rickettsia rickettsii* (relative abundance > 0.1%)

The high-throughput sequencing of the 16S rRNA gene of 37 *H. longicornis* tick samples using iSeq 100 produced an average total read count of 7454 and 72 average number of OTUs from 37 tick samples. At the species level, all samples were dominated by *Coxiella* AB001519 (5.48–89.51% of the total community, average: 39.87%) (Fig. 1). *Williamsia maris* (0.04–41.83% of the total community, average: 14.56%), which was the second most abundant species, was also detected in all samples. *Rickettsia rickettsii* was detected in 16 out of 37 tick samples (Fig. 1).

### Detection of rickettsiae using PCR

**Fig. 2:**
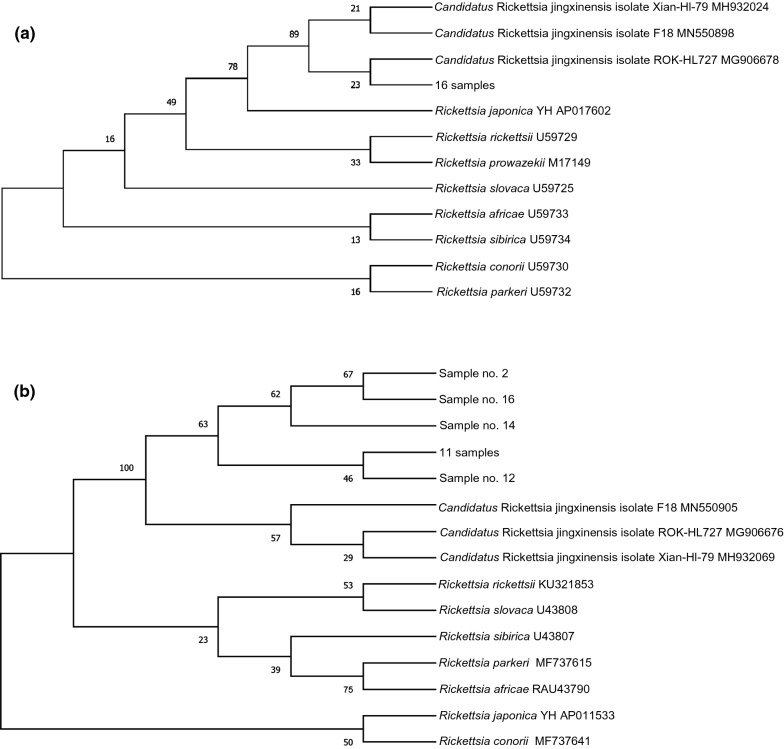
Phylogenetic trees based on partial sequences of *gltA* (**a**) and *ompA* (**b**) genes. Sequences of the *Rickettsia* spp. detected in the present study were aligned with those retrieved from the GenBank database. The phylogenetic trees were constructed in MEGA X software using the maximum-likelihood method, employing the Tamura-nei model of nucleotide substitution with 1000 bootstrap replications

For the same set of samples, nested PCR of the 17 kDa surface protein gene determined 16 samples as positive for rickettsiae; the same samples were positive with NGS using iSeq 100. To identify the strain of *Rickettsia* spp. in our samples, species-specific PCR was performed using primers for partial sequences of *gltA* and *ompA*, and the resulting phylogenetic trees were obtained (Fig. 2). The PCR amplicon sequences of the *gltA* gene of all 16 *Rickettsia*-positive samples in the nested PCR demonstrated 100% homology to previously reported DNA sequences, which are ‘*Candidatus* Rickettsia jingxinensis’ isolate Xian-Hl-79 (MH932024), ‘*Ca*. R. jingxinensis’ isolate F18 (MN550898), and ‘*Ca*. Rickettsia longicornii’ isolate ROK-HL727 (MG906678) (Fig. 2a, Additional file 2: Figure S1). For *ompA*, DNA sequences from *Rickettsia* revealed 95.9–99.5% homology to ‘*Ca*. R. longicornii’ isolate ROK-HL727 (MG906676), ‘*Ca*. R. jingxinensis’ isolate Xian-Hl-79 (MH932069), and ‘*Ca*. R. jingxinensis’ isolate F18 (MN550905) (Fig. 2B, Additional file 3: Figure S2, Additional file 1: Table S1). Among the 16 *Rickettsia-*positive samples, 11 samples had 99.5% identity with those of ‘*Ca*. R. longicornii’ isolate ROK-HL727 (MG906676) and ‘*Ca*. R. jingxinensis’ isolate Xian-Hl-79 (MH932069) and 98.6% identity with the sequence of ‘*Ca*. R. jingxinensis’ isolate F18 (MN550905). Four samples showed relatively lower sequence identity (95.9–98.6%), which also showed poor sequencing quality (double peaks). One sample could not be sequenced (sample 13).

### The association between the presence of *Rickettsia* and the microbiome in ticks

**Fig. 3:**
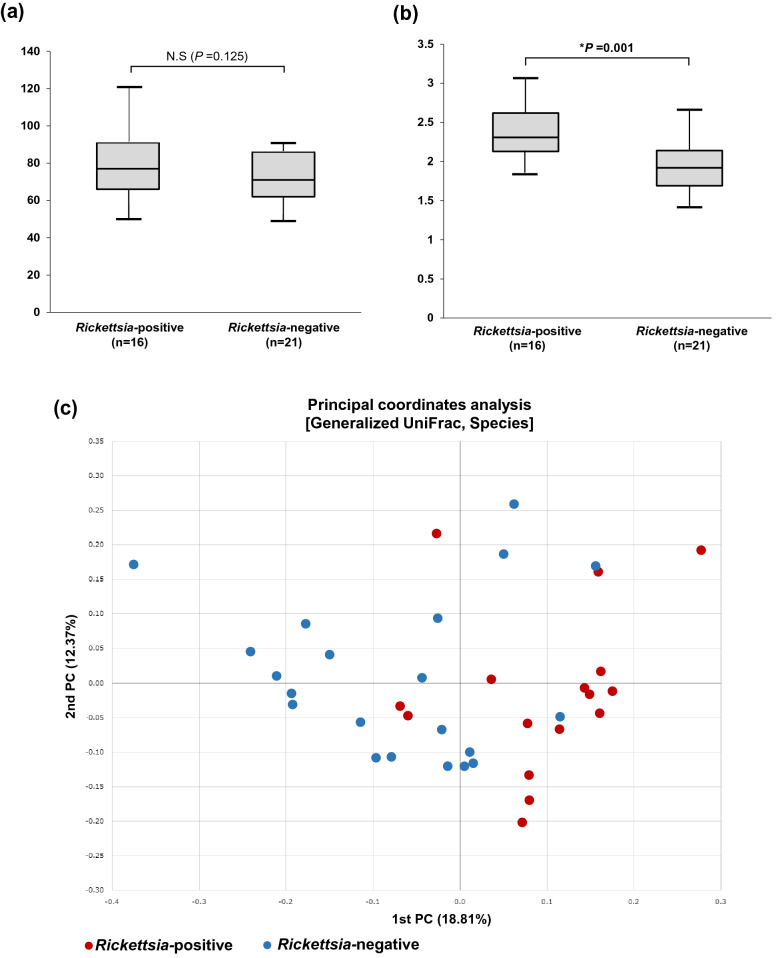
Alpha and beta diversities of the microbiome of *Rickettsia*-positive groups and *Rickettsia-*negative groups. **a** The number of operational taxonomic units (OTUs) and **b** Shannon index between the *Rickettsia-*positive (*n* = 16) and *Rickettsia-*negative (*n* = 21) groups. **c** Microbiome composition of *Rickettsia*-positive and *Rickettsia-*negative groups are shown by principal coordinate analysis (PCoA)

**Fig. 4:**
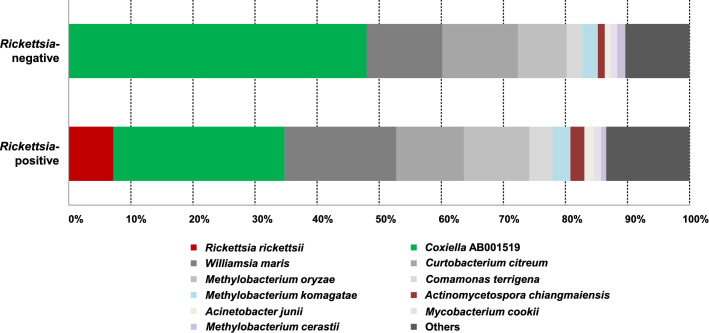
The average microbiome composition of *Rickettsia*-positive ticks (*n* = 16) and *Rickettsia-*negative ticks (*n* = 21) at the species level. Species accounting for than 1% of total reads are shown

**Fig. 5:**
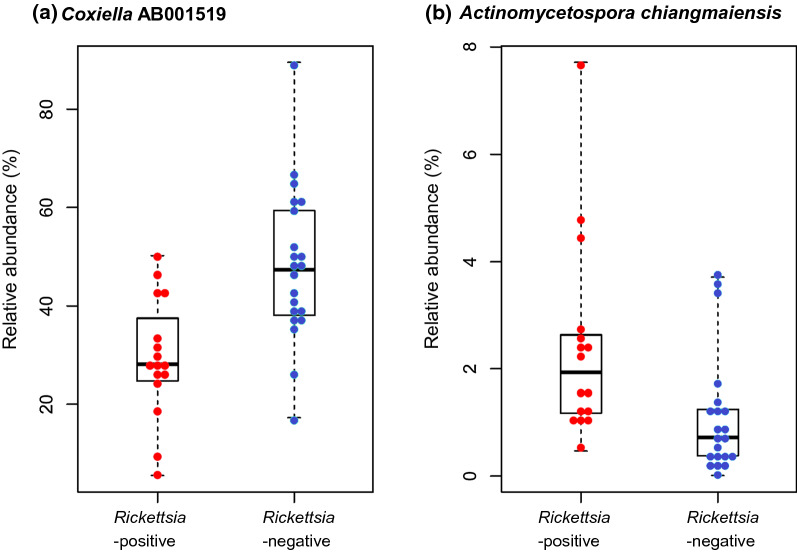
Swarm plots of the relative abundance of **a** *Coxiella* AB001519, and **b** *Actinomycetospora chiangmaiensis* in *Rickettsia*-positive ticks (*n* = 16), and *Rickettsia-*negative ticks (*n* = 21)

We investigated the association between the presence of *Rickettsia* and the microbiome composition in ticks. The average of the total read counts for the *Rickettsia*-positive group (*n* = 16) and the *Rickettsia*-negative group (*n* = 21) were 8033 and 7012, respectively. The number of OTUs were not significantly different between the *Rickettsia*-positive and *Rickettsia*-negative groups (Fig. 3a). However, the Shannon index (bacterial diversity) was significantly different between the two groups (Fig. 3b). The results of PCoA (Fig. 3c) showed that the samples were not well organized as per *Rickettsia* positivity, but in those using permutational multivariate analysis of variance (PERMANOVA), *Rickettsia* positivity was found to be a significant factor in determining the tick microbiome composition (*P* = 0.001). Following this, we analyzed the bacterial composition between the two groups and found that *Coxiella* AB001519 accounted for 29.27% and 48.25% of the total reads in the *Rickettsia-*positive and *Rickettsia*-negative groups, respectively (Fig. 4). The differentially abundant bacterial species between the groups were identified using the ALDEx2 algorithm. The relative abundance of *Coxiella* AB001519 was higher in the *Rickettsia*-negative ticks than *Rickettsia*-positive ticks (the expected *P* value of Wilcoxon rank test = 0.022); conversely, the relative abundance of *Actinomycetospora chiangmaiensis* was higher in the *Rickettsia*-positive ticks than *Rickettsia*-negative ticks (the expected *P* value of Wilcoxon rank test = 0.008). This pattern was also seen in the swarm plots of the relative abundances (Fig. 5a, b).

## Discussion

Ticks can transmit bacterial, parasitic, and viral pathogens (often zoonotically) and often harbor more than one agent simultaneously [36]. Thus, obtaining broader information about pathogens in ticks is important from the perspective of proper diagnosis and treatment of these diseases [37]. In this study, we demonstrated that the recently released NGS instrument, iSeq 100, is useful for screening of bacteria in ticks. NGS approaches have the ability to identify a wide range of known or unknown pathogens or discover new organisms from a single test [19] without the need to design specific primers for each pathogen. This method makes it possible to identify pathogens immediately, not only in ticks, but also in arthropods that serve as vectors and reservoirs for pathogens, such as mosquitoes, tsetse flies, and sand flies.

Considering this advantage, in this study, we screened the tick-borne bacteria using iSeq 100 and found that 16 of the 37 ticks harbored pathogens of *R. rickettsii*. For an accurate taxonomic classification of the species of the detected *Rickettsia* spp., we used conventional PCR to amplify *gltA* and *ompA* sequences of the *Rickettsia* spp. and compared them with sequences deposited in GenBank using BLAST. In the phylogenetic analysis performed using MEGA X software, the sequence similarity to sequences of ‘*Ca*. R. jingxinensis’ isolate Xian-Hl-79, ‘*Ca*. R. jingxinensis’ isolate F18, and ‘*Ca*. R. longicornii’ isolate ROK-HL727 was 100% for *gltA,* indicating a close relationship between rickettsial isolates from *H. longicornis* from Korea with those from other East Asian countries. The close clustering of the Chinese and Korean strains of *Rickettsia* spp. may indicate a close epidemiological link between these strains.

Ixodid ticks (e.g., *H. longicornis*, *H. flava*, *Ixodes persulcatus*, and *I. nipponensis*) in Asia have the potential to be primary vectors/repositories of rickettsiae of medical and veterinary importance [15]. In 2006, the first case in Korea of *R. japonica* was isolated from a spotted fever patient [13]. Recent studies show a high prevalence of the emerging pathogen, *Rickettsia raoultii,* in canine ticks [38].

In our study, the sequences of *gltA* and *ompA* are identical or highly homologous to those of ‘*Ca*. R. jingxinensis’ isolate Xian-Hl-79, ‘*Ca*. R. jingxinensis’ isolate F18, and “*Ca*. R. longicornii’ isolate ROK-HL727. ‘*Ca*. R. jingxinensis,’ a novel *Rickettsia* species in *Rhipicephalus microplus* and *H. longicornis* ticks, was first discovered in China (Shenyang and Wuhan) [39–41] and subsequently reported in Korea (Chungnam, Jeonbuk, and Gwangju) [42, 43]. Many associated ‘*Ca*. R. jingxinensis’ sequences have been deposited in GenBank. Of these, a *gltA* sequence (KU853023) was recovered from a patient, suggesting its potential pathogenicity to humans [44]. The pathogenicity of ‘*Ca*. R. longicornii’ is yet to be determined. However, 99.6% identity was detected with the ‘*Ca*. R. longicornii’ *ompA* sequence of an isolate from rodent spleen tissue obtained in Korea, and 99.9% identity was detected with the *‘Ca*. R. longicornii’ *gltA* sequence of an isolate from a human blood sample obtained in China [18, 44]. These results suggest that ‘*Ca*. R. longicornii’ has the potential to infect mammalian hosts, including humans [45].

Although not detected in our samples, *Borrelia burgdorferi*, *B. afzelii*, and *B. garinii,* which are implicated in Lyme disease, have been isolated from *Ixodes persulcatus*, *I. nipponensis*, and *I. granulatus*, which are distributed in parts of Korea [46, 47]. In addition, several Lyme disease (borreliosis) cases have been reported in Korea [48–51].

*Coxiella* AB001519, known as a *Coxiella*-like symbiont of *H. longicornis*, was detected in all 37 samples [52]. A bacterium belonging to *Coxiella* was reported as the primary endosymbiont of *Amblyomma americanum* ticks and was found to improve the reproductive health of the ticks [53–55]. *Coxiella* AB001519 was first identified in Japan in a phylogenetic association with the *Coxiella*-like endosymbiont of *H. longicornis*, and the *Coxiella*-like endosymbiont presenting more than 99% homology with *Coxiella* AB001519 in Thailand, China, and Korea was found in *H. longicornis* [56–58].

In the present study, the relative abundance of *A. chiangmaiensis* found to be significantly higher in *Rickettsia* spp.-positive samples than in *Rickettsia* spp.-negative samples. *A. chiangmaiensis*, a Gram-positive bacterium, was isolated from the soil of a tropical rainforest in northern Thailand in 2008 [59]. *Actinomycetospora* spp. are commonly found in the environment and are known to infect ticks [60].

Although we confirmed the ability of iSeq 100 to detect potential pathogens using PCR, the rarefaction curves of some samples did not show a plateau in their read counts (Additional file 4: Figure S3). To cover the whole microbial diversity, sufficient reads should be sequenced. To this end, it would be helpful to load a small number of samples in one run or to use a machine with a larger sequencing capacity, such as MiSeq.

The targeted NGS using the bacterial 16S rRNA V4 region in this study cannot identify the exact strain of pathogens. However, this technique can theoretically detect all potentially pathogenic taxa in samples including *Rickettsia* spp., *Coxiella* spp., *Borrelia* spp., *Bartonella* spp., *Francisella* spp., and *Anaplasma* spp., and simultaneously analyze 96 samples. The cost of iSeq 100 reagents required for one run is approximately US $2000, and the sequencing takes 18 h to complete. Therefore, we believe that this method is useful in screening pathogens in arthropod vectors such as ticks, and can be followed by subsequent experiments to identify the exact strain of the suspected pathogens.

## Conclusions

In this study, iSeq 100 was used to investigate the microbiome of *H. longicornis* and the potentially pathogenic *Rickettsia* strain was detected in 16 out of 37 ticks. We believe that this approach can be used for large-scale pathogen screening of arthropods, which can be used in vector-borne disease control programs.

## Supplementary Information

**Additional file 1: Table S1.** Homology of *ompA* gene sequence between the PCR amplicon and the gene of three known *Rickettsia* spp.

**Additional file 2: Figure S1.** The PCR amplicon sequence alignment of *gltA* gene nucleotide sequences from *Rickettsia* spp.

**Additional file 3: Figure S2.** The PCR amplicon sequence alignment of the *ompA* gene nucleotide sequences from *Rickettsia* spp.

**Additional file 4: Figure S3.** Rarefaction curves for the number of operational taxonomic units (OTUs) of the 37 tick samples.

## Data Availability

Raw sequence data are available in NCBI GenBank under BioProject PRJNA728802.

## Abbreviations

SFTS: Severe fever with thrombocytopenia syndrome
NGS: Next-generation sequencing
PCoA: Principal coordinate analysis
ALDEx2: ANOVA-like differential expression version 2
OTUs: Operational taxonomic units
PERMANOVA: Permutational multivariate analysis of variance
LDA: Linear discriminant analysis
