# Supplementary material for: iSeq 100 for metagenomic pathogen screening in ticks
Source: Parasit Vectors. 2021 Jun 29;14:346. doi: 10.1186/s13071-021-04852-w (PMC8244152; doi:10.1186/s13071-021-04852-w)
Supplement: Supplementary file 1 — Additional file 1: Table S1. Homology of ompA gene sequence between the PCR amplicon and the gene of three known Rickettsia spp. [file 13071_2021_4852_MOESM1_ESM.docx]

Additional file 1: Table S1. Homology of *ompA* gene sequence between the PCR amplicon and the gene of three known *Rickettsia* spp.

| Sample  No. | *Candidatus* R. longicornii isolate ROK-HL727  (MG906676) | *Candidatus* R. jingxinensis isolate Xian-Hl-79 (MH932069) | *Candidatus* R. jingxinensis isolate F18  (MN550905) |
| --- | --- | --- | --- |
| 1 | 99.5 | 99.5 | 98.6 |
| 2 | 98.6 | 98.6 | 97.7 |
| 3 | 99.5 | 99.5 | 98.6 |
| 4 | 99.5 | 99.5 | 98.6 |
| 5 | 99.5 | 99.5 | 98.6 |
| 6 | 99.5 | 99.5 | 98.6 |
| 7 | 99.5 | 99.5 | 98.6 |
| 8 | 99.5 | 99.5 | 98.6 |
| 9 | 99.5 | 99.5 | 98.6 |
| 10 | 99.5 | 99.5 | 98.6 |
| 11 | 99.5 | 99.5 | 98.6 |
| 12 | 98.6 | 98.6 | 98.6 |
| 14 | 98.6 | 98.6 | 97.7 |
| 15 | 99.5 | 99.5 | 98.7 |
| 16 | 96.8 | 96.9 | 95.9 |
