# Supplementary material for: iSeq 100 for metagenomic pathogen screening in ticks
Source: Parasit Vectors. 2021 Jun 29;14:346. doi: 10.1186/s13071-021-04852-w (PMC8244152; doi:10.1186/s13071-021-04852-w)
Supplement: Supplementary file 3 — Additional file 3: Figure S2. The PCR amplicon sequence alignment of the ompA gene nucleotide sequences from Rickettsia spp. [file 13071_2021_4852_MOESM3_ESM.pdf]

```
sample_no.1     TGCTGATTATACTATCACTGCAGATGTAGTCGATCGTATTATTACGGCTATAAATGTTGCGGGTACTACTCCCGTAGGTCTAAATATTGCTCAAAATACCGTCGTTGGTTCGATTATAACTAGAGGTAACTTGTTGCCTGTTACTATTGC    150
sample_no.2     TGCTGATTATACTATCACTGCAGATGTAGTCCATCGTATTATTACGGCTATAAATGTTGCGGGTACTACTCCCATAGGTCTAAATATTGCTCAAAATACCGTCGTTGGTTCGATTATAACTAGAGGTAACTTGTTGCCTGTTACTATTGC    150
sample_no.3     TGCTGATTATACTATCACTGCAGATGTAGTCGATCGTATTATTACGGCTATAAATGTTGCGGGTACTACTCCCGTAGGTCTAAATATTGCTCAAAATACCGTCGTTGGTTCGATTATAACTAGAGGTAACTTGTTGCCTGTTACTATTGC    150
sample_no.4     TGCTGATTATACTATCACTGCAGATGTAGTCGATCGTATTATTACGGCTATAAATGTTGCGGGTACTACTCCCGTAGGTCTAAATATTGCTCAAAATACCGTCGTTGGTTCGATTATAACTAGAGGTAACTTGTTGCCTGTTACTATTGC    150
sample_no.5     TGCTGATTATACTATCACTGCAGATGTAGTCGATCGTATTATTACGGCTATAAATGTTGCGGGTACTACTCCCGTAGGTCTAAATATTGCTCAAAATACCGTCGTTGGTTCGATTATAACTAGAGGTAACTTGTTGCCTGTTACTATTGC    150
sample_no.6     TGCTGATTATACTATCACTGCAGATGTAGTCGATCGTATTATTACGGCTATAAATGTTGCGGGTACTACTCCCGTAGGTCTAAATATTGCTCAAAATACCGTCGTTGGTTCGATTATAACTAGAGGTAACTTGTTGCCTGTTACTATTGC    150
sample_no.7     TGCTGATTATACTATCACTGCAGATGTAGTCGATCGTATTATTACGGCTATAAATGTTGCGGGTACTACTCCCGTAGGTCTAAATATTGCTCAAAATACCGTCGTTGGTTCGATTATAACTAGAGGTAACTTGTTGCCTGTTACTATTGC    150
sample_no.8     TGCTGATTATACTATCACTGCAGATGTAGTCGATCGTATTATTACGGCTATAAATGTTGCGGGTACTACTCCCGTAGGTCTAAATATTGCTCAAAATACCGTCGTTGGTTCGATTATAACTAGAGGTAACTTGTTGCCTGTTACTATTGC    150
sample_no.9     TGCTGATTATACTATCACTGCAGATGTAGTCGATCGTATTATTACGGCTATAAATGTTGCGGGTACTACTCCCGTAGGTCTAAATATTGCTCAAAATACCGTCGTTGGTTCGATTATAACTAGAGGTAACTTGTTGCCTGTTACTATTGC    150
sample_no.10    TGCTGATTATACTATCACTGCAGATGTAGTCGATCGTATTATTACGGCTATAAATGTTGCGGGTACTACTCCCGTAGGTCTAAATATTGCTCAAAATACCGTCGTTGGTTCGATTATAACTAGAGGTAACTTGTTGCCTGTTACTATTGC    150
sample_no.11    TGCTGATTATACTATCACTGCAGATGTAGTCGATCGTATTATTACGGCTATAAATGTTGCGGGTACTACTCCCGTAGGTCTAAATATTGCTCAAAATACCGTCGTTGGTTCGATTATAACTAGAGGTAACTTGTTGCCTGTTACTATTGC    150
sample_no.12    TGCTGATTATACTATCACTGCAGATGTAGTCGATCGCATTATTACGGCTATAAATGTTGCGGGTACTACTCCCGTAGGGCTAAATATTGCTCAAAATACCGTCGTTGGTTCGATTATAACTAGAGGTAACTTGTTGCCTGTTACTATTGC    150
sample_no.14    TGCTGATTATACTATCACTGCAGATGTAGTCGATCGTATTATTACGGCTATAAATGTTGCGGGTACTACTCCCATATGTCTAAATATTGCTCAAAATACCGTCGTTGGTTCGATTATAACTAGAGGTAACTTGTTGCCTGTTACTATTGC    150
sample_no.15    TGCTGATTATACTATCACTGCAGATGTAGTCGATCGTATTATTACGGCTATAAATGTTGCGGGTACTACTCCCGTAGGTCTAAATATTGCTCAAAATACCGTCGTTGGTTCGATTATAACTAGAGGTAACTTGTTGCCTGTTACTATTGC    150
sample_no.16    TGCTGATTATACTATCTCTGCAGATGGATTCCATCGTATCATTACGGCTATAAATGTTGCGGGTACTACTCCCATAGGTCTAAATATTGCTCAAAATACCGTCGTTGGTTCGATTATAACTAGAGGTAACTTGTTGCCTGTTACTATTGC    150
                **************** ********* * ** **** ** ********************************* ** * ***********************************************************************


sample_no.1     TGGCAAAAGCTTAACTTTAAACGGTACTAATGCTGTTGCTGCAAATCATGGTTTTGATGCTCCGGCCGATAC    222
sample_no.2     TGGCAAAAGCTTAACTTTAAACGGTACTAATGCTGTTGCTGCAAATCATGGTTTTGATGCTCCGGCCGATAC    222
sample_no.3     TGGCAAAAGCTTAACTTTAAACGGTACTAATGCTGTTGCTGCAAATCATGGTTTTGATGCTCCGGCCGATAC    222
sample_no.4     TGGCAAAAGCTTAACTTTAAACGGTACTAATGCTGTTGCTGCAAATCATGGTTTTGATGCTCCGGCCGATAC    222
sample_no.5     TGGCAAAAGCTTAACTTTAAACGGTACTAATGCTGTTGCTGCAAATCATGGTTTTGATGCTCCGGCCGATAC    222
sample_no.6     TGGCAAAAGCTTAACTTTAAACGGTACTAATGCTGTTGCTGCAAATCATGGTTTTGATGCTCCGGCCGATAC    222
sample_no.7     TGGCAAAAGCTTAACTTTAAACGGTACTAATGCTGTTGCTGCAAATCATGGTTTTGATGCTCCGGCCGATAC    222
sample_no.8     TGGCAAAAGCTTAACTTTAAACGGTACTAATGCTGTTGCTGCAAATCATGGTTTTGATGCTCCGGCCGATAC    222
sample_no.9     TGGCAAAAGCTTAACTTTAAACGGTACTAATGCTGTTGCTGCAAATCATGGTTTTGATGCTCCGGCCGATAC    222
sample_no.10    TGGCAAAAGCTTAACTTTAAACGGTACTAATGCTGTTGCTGCAAATCATGGTTTTGATGCTCCGGCCGATAC    222
sample_no.11    TGGCAAAAGCTTAACTTTAAACGGTACTAATGCTGTTGCTGCAAATCATGGTTTTGATGCTCCGGCCGATAC    222
sample_no.12    TGGCAAAAGCTTAACTTTAAACGGTACTAATGCTGTTGCTGCAAATCATGGTTTTGATGCTCCGGCCGATAC    222
sample_no.14    TGGCAAAAGCTTAACTTTAAACGGTACTAATGCTGTTGCTGCAAATCATGGTTTTGATGCTCCGGCCGATAC    222
sample_no.15    TGGCAAAAGCTTAACTTTAAACGGTACTAATGCTGTTGCTGCAAATCATGGTTTTGATGCTCCGGCCGATAC    222
sample_no.16    TGGCAAAAGCTTAACTTTAAACGGTACTAATGCTGTTGCTGCAAATCATGGTTTTGATGCTCCGGCCGATAC    222
                ************************************************************************
```
