# Supplementary figures and images for: iSeq 100 for metagenomic pathogen screening in ticks
Source: Parasit Vectors. 2021 Jun 29;14:346. doi: 10.1186/s13071-021-04852-w (PMC8244152; doi:10.1186/s13071-021-04852-w)

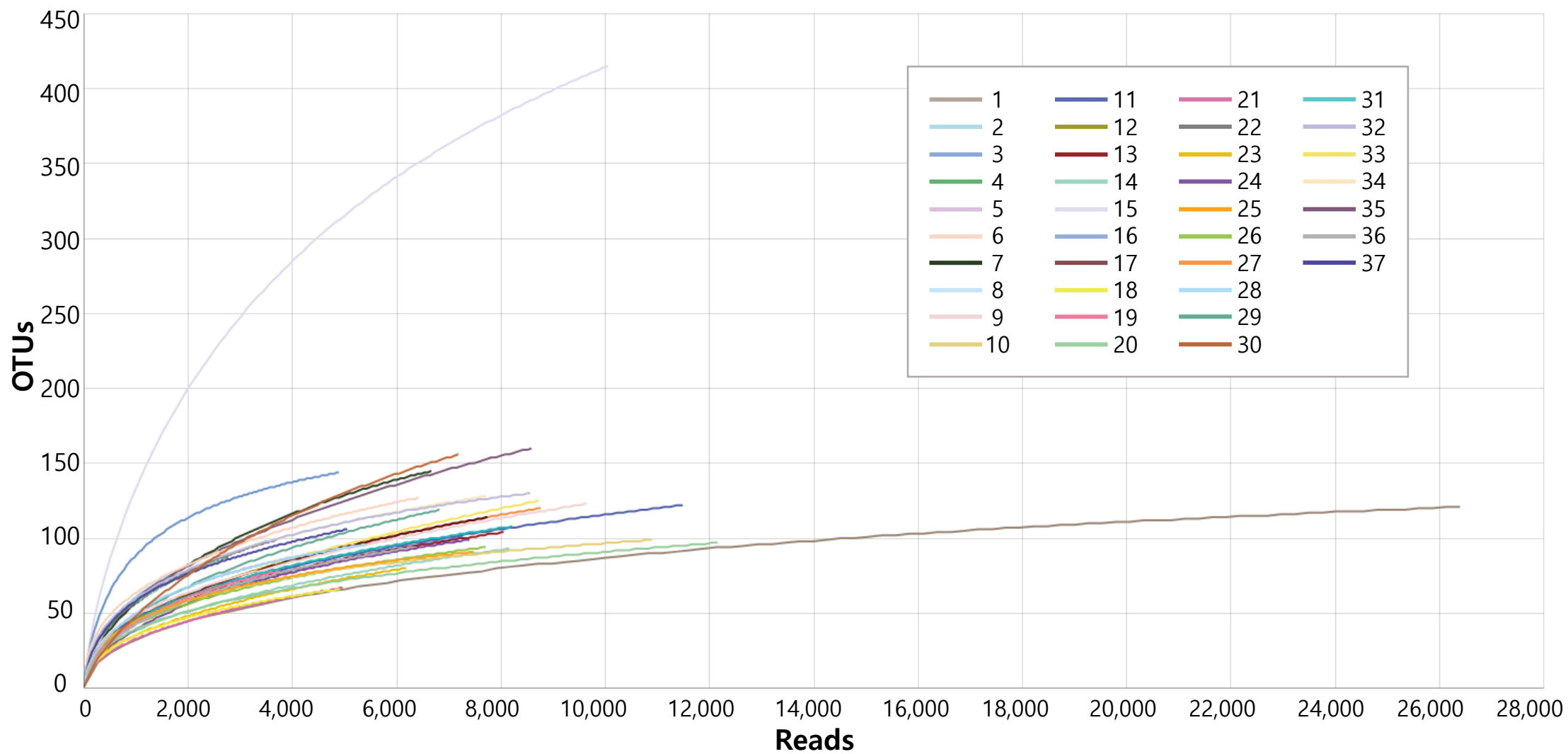

Supplement: Supplementary file 4 — Additional file 4: Figure S3. Rarefaction curves for the number of operational taxonomic units (OTUs) of the 37 tick samples. [file 13071_2021_4852_MOESM4_ESM.pdf]
